# Construction and validation of a COVID-19 pandemic trend forecast model based on Google Trends data for smell and taste loss

**DOI:** 10.3389/fpubh.2022.1025658

**Published:** 2022-12-01

**Authors:** Jingguo Chen, Hao Mi, Jinyu Fu, Haitian Zheng, Hongyue Zhao, Rui Yuan, Hanwei Guo, Kang Zhu, Ya Zhang, Hui Lyu, Yitong Zhang, Ningning She, Xiaoyong Ren

**Affiliations:** ^1^Department of Otorhinolaryngology-Head and Neck Surgery, The Second Affiliated Hospital of Xi'an Jiaotong University, Xi'an, China; ^2^School of Computer Science and Technology, Xi'an Jiaotong University, Xi'an, China; ^3^School of Mathematics and Statistics, Xi'an Jiaotong University, Xi'an, China; ^4^Health Science Center, Xi'an Jiaotong University, Xi'an, China

**Keywords:** COVID-19, big data, smell, taste, prediction

## Abstract

**Aim:**

To explore the role of smell and taste changes in preventing and controlling the COVID-19 pandemic, we aimed to build a forecast model for trends in COVID-19 prediction based on Google Trends data for smell and taste loss.

**Methods:**

Data on confirmed COVID-19 cases from 6 January 2020 to 26 December 2021 were collected from the World Health Organization (WHO) website. The keywords “loss of smell” and “loss of taste” were used to search the Google Trends platform. We constructed a transfer function model for multivariate time-series analysis and to forecast confirmed cases.

**Results:**

From 6 January 2020 to 28 November 2021, a total of 99 weeks of data were analyzed. When the delay period was set from 1 to 3 weeks, the input sequence (Google Trends of loss of smell and taste data) and response sequence (number of new confirmed COVID-19 cases per week) were significantly correlated (*P* < 0.01). The transfer function model showed that worldwide and in India, the absolute error of the model in predicting the number of newly diagnosed COVID-19 cases in the following 3 weeks ranged from 0.08 to 3.10 (maximum value 100; the same below). In the United States, the absolute error of forecasts for the following 3 weeks ranged from 9.19 to 16.99, and the forecast effect was relatively accurate. For global data, the results showed that when the last point of the response sequence was at the midpoint of the uptrend or downtrend (25 July 2021; 21 November 2021; 23 May 2021; and 12 September 2021), the absolute error of the model forecast value for the following 4 weeks ranged from 0.15 to 5.77. When the last point of the response sequence was at the extreme point (2 May 2021; 29 August 2021; 20 June 2021; and 17 October 2021), the model could accurately forecast the trend in the number of confirmed cases after the extreme points. Our developed model could successfully predict the development trends of COVID-19.

**Conclusion:**

Google Trends for loss of smell and taste could be used to accurately forecast the development trend of COVID-19 cases 1–3 weeks in advance.

## Introduction

COVID-19 has ravaged countries worldwide, seriously threatening human life and health and causing severe damage to the social order and economic development (1). Governments in all countries attach great importance to pandemic prevention and control, and pandemic trend forecasting is critical to this end.

Big data from the Internet played an essential role in pandemic monitoring and prevention, disease source tracing, drug screening, medical treatment, product recovery, and other applications (2–4). Based on Internet big data, such as Google Trends and Baidu Trends, the occurrence and development of infectious disease trends can be predicted (5, 6). Previous studies have confirmed a significant positive correlation between Google Trends data for smell and taste loss and the daily number of confirmed COVID-19 cases (7–10).

Previous studies have found that loss of smell and taste is an early symptom of COVID-19 infection and can serve as a reliable indicator in COVID-19 diagnosis (11, 12). Most clinical symptoms in patients with COVID-19 who have olfactory and gustatory disorders are not serious, so these patients are difficult to diagnose in a timely fashion, raising the risk for the spread of infection. However, patients with olfactory and gustatory disorders usually search for information and methods to deal with smell and taste loss online. Therefore, analysis of big data for information on smell and taste loss retrieved from the Internet can likely provide an essential reference for pandemic prevention and control. By analyzing billions of Google search results worldwide, Google Trends displays the search volume and relevant statistical data for each keyword entered into Google, which can reflect the scale, timeliness, accuracy, and intuitiveness of the data. In this study, we used Google Trends data on smell and taste loss, as well as the daily pandemic statistics reported by the World Health Organization (WHO), to build a COVID-19 global pandemic trend forecast model. Our study can provide an essential scientific basis for the prevention and control of COVID-19.

## Research data and methods

### Raw data

#### Number of confirmed COVID-19 cases

Using the WHO official website (https://covid19.who.int/info), we downloaded daily data on newly confirmed cases of COVID-19 infection from 6 January 2020 to 26 December 2021 in the data module. We then aggregated these to obtain the weekly number of new confirmed cases worldwide, in the United States (US), and in India.

#### Google Trends data on smell and taste loss

Using the Google Trends platform (https://trends.google.com), we used “loss of smell” and “loss of taste” as keywords to obtain Google Trends data on loss of smell and taste worldwide, in the United States, and in India from 6 January 2020 to 26 December 2021.

### Data preprocessing

#### Normalization of confirmed case data

Because the maximum retrieval volume defined by Google Trends is 100, we normalized the maximum number of weekly new cases to 100 such that the weekly confirmed cases data were distributed within the range of 0–100.

#### Outliers

Due to the potential influence of media reports or other factors, there may be abnormal changes in the Google Trends data for individual weeks, which would adversely affect the analysis of the overall trend for loss of smell and taste; therefore, we defined outliers.

For the detection of outliers, the following judgment principles were used:

For a given time series {*N*_*t*_}, if


(1) $$\begin{array}{l} \frac{1}{t}\sum_{j=1}^{t}N_{j}-6\sqrt{\bar{N_{t}^{2}}-{\bar{N_{t}}}^{2}}<N_{t+1}<\frac{1}{t}\sum_{j=1}^{t}N_{j}\\ \;+6\sqrt{\bar{N_{t}^{2}}-{\bar{N_{t}}}^{2}} \end{array}$$

This means that *N*_*t*+1_ is not an outlier. Otherwise, it can be concluded that *N*_*t*+1_ is an outlier.

For some outliers, we used the linearization method for modification. Assuming *N*_*i*_, *N*_*i*+1_, ⋯ , *N*_*i*+*k*−2_, *N*_*i*+*k*−1_were k adjacent outlier points, we first calculated a straight line through two points (*i*−1, *N*_*i*−1_), (*i*+*k, N*_*i*+*k*_) and then replaced the k outlier points with corresponding equally spaced points on the straight line.

### Calculation of cross-correlation function (CCF) between the input sequence and response sequence

We analyzed the CCF of input sequences (Google Trends data for loss of smell and Google Trends data for loss of taste) and response sequences (number of new confirmed cases per week during the COVID-19 pandemic) to determine the lag effect of Google Trends on the development trend of the COVID-19 pandemic.

The calculation method of the CCF was as follows.

For the sample {*U*_*t*_, *t* = 1, 2, ⋯ , *n*}, {*V*_*t*_, *t* = 1, 2, ⋯ , *n*} of time series {*U*_*t*_}, {*V*_*t*_}, we calculated the interaction covariance function of the sample as an estimate of the interaction covariance function of {*U*_*t*_}, {*V*_*t*_}:


(2) $$\begin{array}{l} C_{uv\;}\left(k\right)=\frac{1}{n}\overset{n-k}{\underset{t=1}{\sum }}\left(U_{t}-\bar{U}\right)\left(V_{t+k}-\bar{V}\right),\;k=0,1,2\cdots \end{array}$$

Similarly, the sample CCF can be regarded as follows:


(3) $$\begin{array}{l} \gamma_{uv\;}\left(k\right)=\frac{C_{uv}\left(k\right)}{S_{u}S_{v}},\;k=0,1,2\cdots \end{array}$$

where *S*_*u*_ is the sample standard deviation of *U*_*t*_; *S*_*v*_ is the sample standard deviation of *V*_*t*_.

### Transfer function model fitting

We adopted the Box–Jenkins iterative three-stage modeling approach, namely, identification, estimation, and diagnostic checking.

(1) Structure of the model

We denoted the Google Trends search volume for “loss of smell” or “loss of taste” in 1 week as *X*_*t*_ and the number of newly diagnosed COVID-19 cases (normalized) as *Y*_*t*_; then, the structure of the transfer function model is given as follows:


(4) $$\begin{array}{l} Y_{t}=\frac{\omega \left(B\right)B^{b}}{\delta \left(B\right)}X_{t}+\frac{\theta \left(B\right)}{\varphi \left(B\right)}a_{t} \end{array}$$

where *B* is the backshift operator, $w\left(B\right)=w_{0}-\overset{s}{\underset{i=1}{\sum }}w_{i}B^{i}$,$\delta \left(B\right)=1-\overset{r}{\underset{i=\;1}{\sum }}\delta_{i}B^{i}$

φ(*B*) is the autoregressive polynomial, $\varphi \left(B\right)=1-\overset{p}{\underset{i=\;1}{\sum }}\varphi_{i}B^{i}$

θ(*B*) is the moving average polynomial, $\theta \left(B\right)=1-\overset{q}{\underset{i=\;1}{\sum }}\theta_{i}B^{i}$

*a*_*t*_ is the white noise process, and *b* is the lag period of *X*_*t*_.

(2) Identification of the model

Because both *X*_*t*_ and *Y*_*t*_ are non-stationary sequences, and regressions with non-stationary series are spurious and the analyses are not valid, we applied the first-order difference transformation to obtain the stationary sequences.


(5) $$\begin{array}{l} Z_{t}^{\left(x\right)}=\nabla X_{t} \end{array}$$

(6) $$\begin{array}{l} Z_{t}=\nabla Y_{t} \end{array}$$

**Figure 1:**
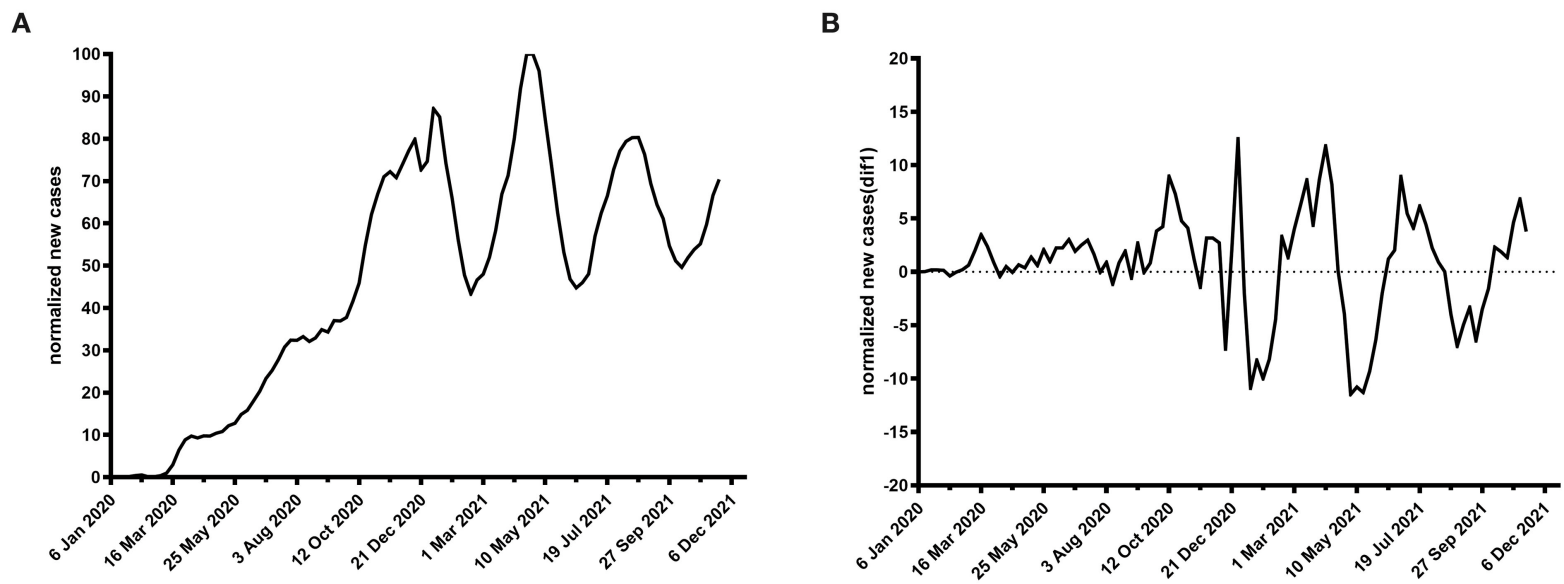
Time plot of normalized newly confirmed COVID-19 cases per week worldwide. **(A)** Raw data, **(B)** first-order difference.

**Figure 2:**
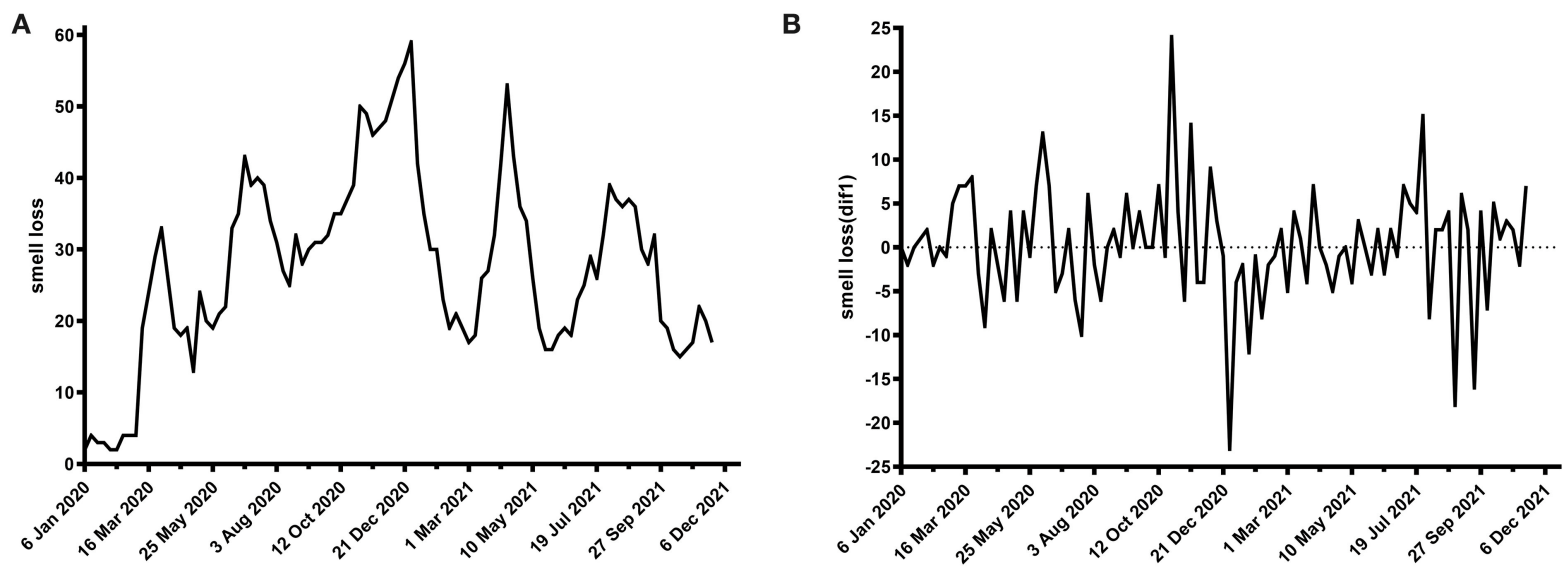
Time plot of Google Trends for smell loss per week worldwide. **(A)** Raw data, **(B)** first-order difference.

Some cases are shown in Figures 1, 2.

Therefore, the structure of the model can be transformed into the following equation:


(7) $$\begin{array}{l} Z_{t}=\frac{\omega \left(B\right)B^{b}}{\delta \left(B\right)}Z_{t}^{\left(x\right)}+\frac{\theta \left(B\right)}{\varphi \left(B\right)}a_{t} \end{array}$$

Then, we conducted pre-white noise processing on $Z_{t}^{\left(x\right)}$ and *Z*_*t*_. Next, by observing the features of a CCF diagram of $Z_{t}^{\left(x\right)}$ and *Z*_*t*_, the values *b, s, r* could be determined, and then, *w*(*B*) and δ(*B*) could be calculated. After that, it is necessary to identify the white noise property of the residual. If the residual is a white noise sequence, meaning that there is no useful information to further extract, the transfer function model has been established; otherwise, if the residual is a non-white noise sequence, the autoregressive integrated moving average (ARIMA) model should be used to extract the information. The orders of AR and MA parameters can be identified by examining the autocorrelation and partial autocorrelation function; then, θ(*B*)and φ(*B*) are calculated to obtain the transfer function model.

(3) Parameter estimation: Parameters were estimated using the non-linear least-squares method.
(4) Model diagnosis was done using the following:

① Significance test of parameters.

② Autocorrelation check of residuals.

③ Cross-correlation check of residuals with the input sequence.

## Results

### Lag effect of Google Trends for smell and taste loss during the COVID-19 pandemic

**Figure 3:**
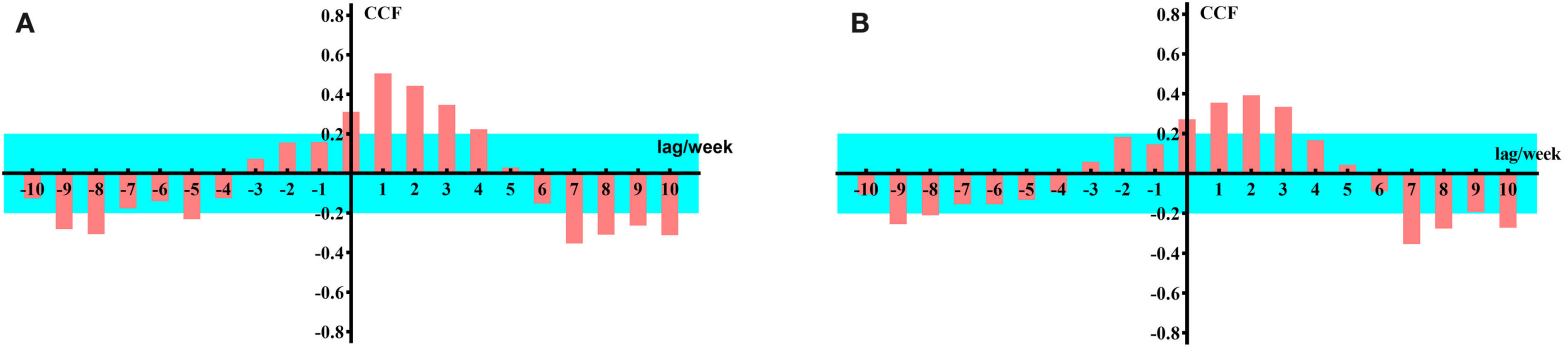
Cross-correlation function values for global data. **(A)** Google Trends data for loss of smell worldwide as the input sequence. **(B)** Google Trends data for loss of taste worldwide as the input sequence.

First, we selected the Google Trends data for anosmia and ageusia worldwide from 6 January 2020 to 28 November 2021 as the input sequence and the weekly new confirmed cases during the same period as the response sequence. Then, by calculating the CCF between the input sequence and response sequence when the former lagged in different weeks, we could analyze the lag effect of the input sequence on the response sequence. The results are shown in Figure 3.

The unit of the horizontal axis in the figure is 1 week, which represents the number of lag periods of the input sequence; the vertical axis represents the CCF. The blue background represents a two-standard error interval. If the CCF is outside the two-standard errors when the input sequence is lagged by k weeks, it can be concluded that the CCF is significantly changed (*P* < 0.001), which means that the input sequence {*U*_*t*_} and response sequence {*V*_*t*+*k*_} are significantly correlated (*P* < 0.05).

**Figure 4:**
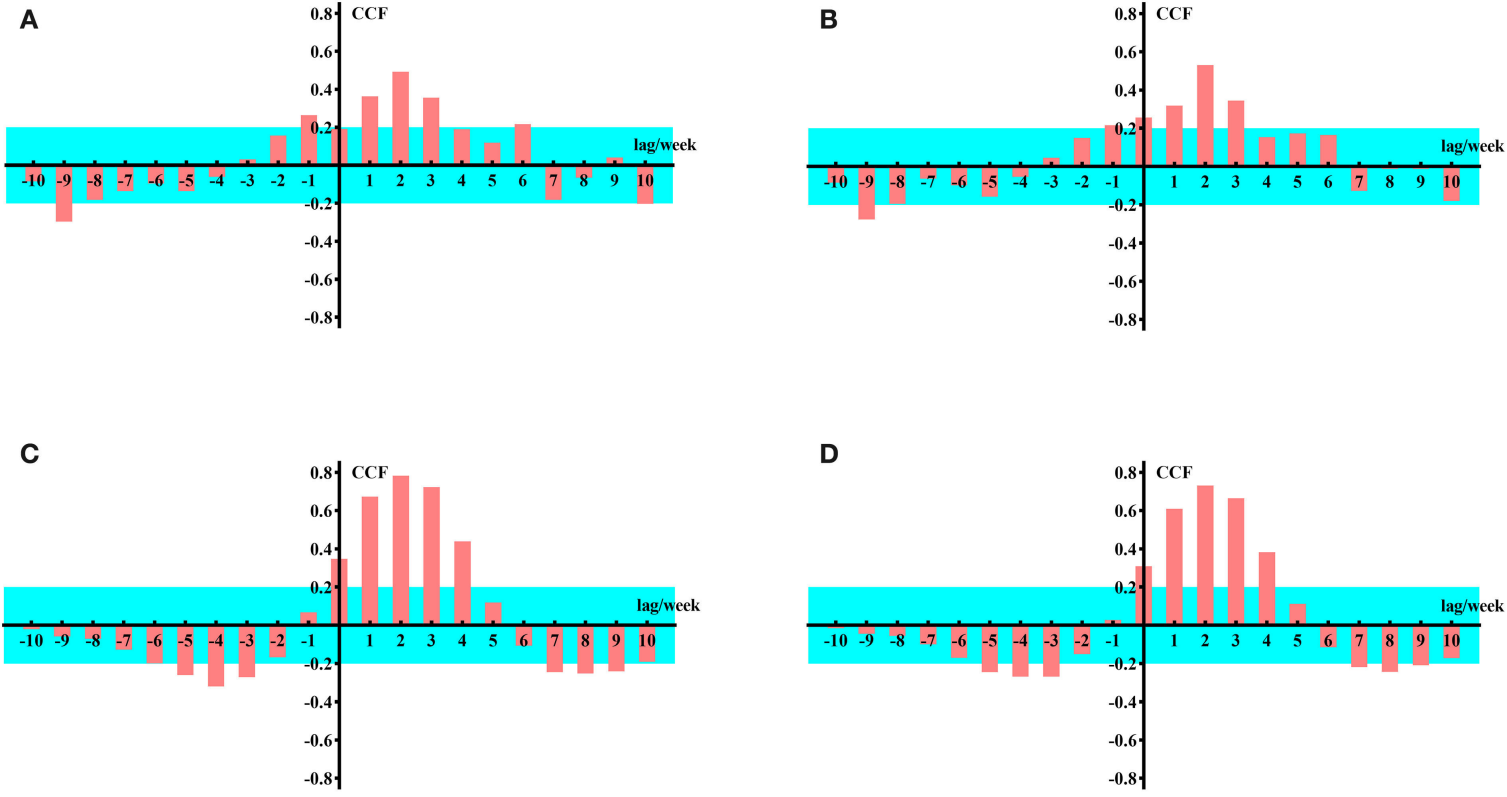
Cross-correlation function values for data from the United States and India. **(A)** Google Trends data for United State loss of smell as the input sequence. **(B)** Google Trends data for United State loss of taste as the input sequence. **(C)** Google Trends data for loss of smell in India as the input sequence. **(D)** Google Trends data for loss of taste in India as the input sequence.

From Figure 3, it can be concluded that when k = 1–3, the input sequence is significantly correlated with the response sequence; therefore, on a global scale, the impact of Google Trends for anosmia and ageusia on the weekly number of newly confirmed cases of COVID-19 lags by 1–3 weeks. Similarly, we can draw the same conclusion from data from the United States and India (Figure 4).

The CCF of the input and response sequence with a lag of 1–3 weeks is shown in Table 1.

**Table 1:** Results of cross-correlation function analysis.

**Area**	**Input sequence**	**Calculation results of cross-correlation function**					
		**Lag 1**		**Lag 2**		**Lag 3**	
		**CCF**	***P*-value**	**CCF**	***P*-value**	**CCF**	***P*-value**
Global	Google Trends of anosmia	0.51	<0.001	0.44	<0.001	0.35	<0.001
	Google Trends of ageusia	0.36	<0.001	0.39	<0.001	0.33	<0.001
US	Google Trends of anosmia	0.36	<0.001	0.49	<0.001	0.35	<0.001
	Google Trends of ageusia	0.32	0.001	0.53	<0.001	0.35	<0.001
India	Google Trends of anosmia	0.67	<0.001	0.78	<0.001	0.72	<0.001
	Google Trends of ageusia	0.61	<0.001	0.73	<0.001	0.66	<0.001

### Construction and accuracy test of the COVID-19 forecast model

#### Construction of the transfer function model

We selected 99 weeks of data from 6 January 2020 to 28 November 2021 with a focus on the whole world, the United States, and India as the raw data for modeling. Taking the weekly number of newly confirmed COVID-19 cases (normalized) in the corresponding region as the response sequence and Google Trends data for anosmia and ageusia as the input sequence, we established the corresponding transfer function models and calculated the parameters in the models. Model construction and parameter calculation were implemented using SAS 9.4 (SAS Institute Inc., Cary, NC, USA). The results are shown in Table 2.

**Table 2:** Transfer function models for different areas and different input sequences.

**Input sequence**	**Area**	**Transfer function**	**Residual**
Google Trends of anosmia	Global	$\frac{0.24505-0.89657B-0.03937B^{2}}{1-1.71231B+0.82890B^{2}}B$	$\frac{1}{1-0.47913B}\alpha_{t}$
	US	$\frac{0.28733-0.92108B^{2}}{1-1.52293B+0.55607B^{2}}B$	(1+0.21552*B*)α_*t*_
	India	$\frac{0.18375-0.51241B^{2}}{1-0.77260B+0.39383B^{2}}B^{3}$	$\frac{1+0.43898B}{1-0.73118B}\alpha_{t}$
Google Trends of ageusia	Global	$\frac{0.10155-1.42692B^{2}}{1-1.74075B+0.85038B^{2}}B^{2}$	$\frac{1+0.45126B}{1-0.25973B}\alpha_{t}$
	US	$\frac{0.35544-0.88787B^{2}}{1-1.45079B+0.48766B^{2}}B$	α_*t*_
	India	$\frac{0.09459-0.51358B^{2}}{1-0.93742B^{2}}B^{3}$	$\frac{1}{1-1.44276B+0.69438B^{2}}$ α_*t*_

#### Forecast results

First, using the transfer function model established above, we calculated the weekly number of newly confirmed COVID-19 cases (normalized) from 6 January 2020 to 28 November 2021 globally and in the United States and India. Second, we used the model to calculate the weekly number of new confirmed cases from 29 November 2021 to 26 December 2021, as the forecast of the response sequence for the following 4 weeks. Finally, the 95% confidence interval of the forecast was calculated. The results are shown in Table 3.

**Table 3:** Forecast values and 95% confidence interval for the following 4 weeks.

**Area**	**Input sequence**	**Google Trends of anosmia**			**Google Trends of ageusia**		
	**Week**	**Forecast**	**95% Confidence interval**		**Forecast**	**95% Confidence interval**	
Global	1	73.17	67.45	78.89	73.72	67.71	79.74
	2	75.23	64.73	85.73	77.11	65.20	89.03
	3	76.50	61.37	91.63	79.68	63.17	96.20
	4	76.95	57.47	96.43	81.26	60.88	101.63
US	1	35.08	26.83	43.34	35.73	27.47	43.99
	2	38.26	24.77	51.76	36.58	24.21	48.96
	3	39.88	20.77	58.99	37.05	19.59	54.50
	4	40.57	15.98	65.16	37.41	14.64	60.18
India	1	2.55	−2.08	7.18	1.78	−2.50	6.07
	2	1.94	−9.13	13.00	0.76	−10.55	12.07
	3	0.94	−16.91	18.80	0.59	−19.35	20.52
	4	0.36	−24.33	25.06	0.16	−28.59	28.91

**Figure 5:**
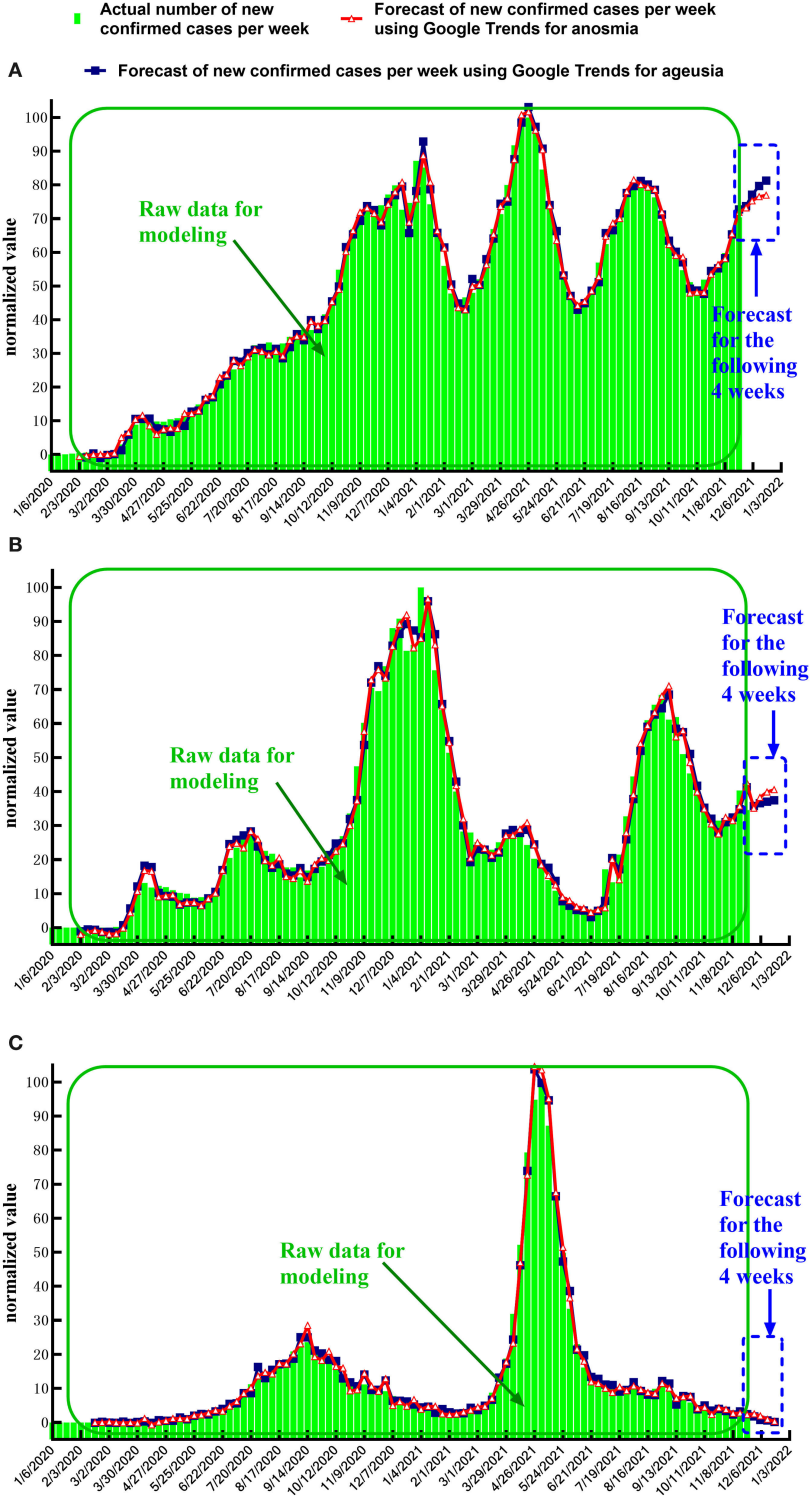
Timing diagram of actual value and forecast value for weekly new cases. **(A)** Global, **(B)** United States, and **(C)** India.

The timing diagram of the actual data and forecast value was plotted using GraphPad Prism 8.0 (GraphPad Software, Inc., San Diego, CA, USA) for data visualization (Figure 5).

#### Accuracy test of the transfer function model

From Figure 5, it can be preliminarily considered that the forecast results are ideal. To test the accuracy of the model, we downloaded the daily number of newly confirmed COVID-19 cases from 29 November 2021 to 26 December 2021 from the official WHO website and aggregated these to obtain the weekly number of new confirmed cases. We then normalized the data to obtain the actual value of the response sequence in the following 4 weeks. Finally, model accuracy was tested by calculating the absolute error between the forecast value and the actual value in the following 4 weeks. The results are shown in Table 4.

**Table 4:** Absolute error between forecast values and actual values for the following 4 weeks.

**Area**	**Input sequence**	**Google Trends of anosmia**			**Google Trends of ageusia**		
	**Week**	**Forecast**	**Actual value**	**Absolute error**	**Forecast**	**Actual value**	**Absolute error**
Global	1	73.17	74.26	1.09	73.72	74.26	0.54
	2	75.23	75.34	0.11	77.11	75.34	1.77
	3	76.50	79.60	3.10	79.68	79.60	0.08
	4	76.95	99.74	22.79	81.26	99.74	18.48
US	1	35.08	44.92	9.84	35.73	44.92	9.19
	2	38.26	49.81	11.55	36.58	49.81	13.23
	3	39.88	54.04	14.17	37.05	54.04	16.99
	4	40.57	84.57	44.00	37.41	84.57	47.16
India	1	2.55	2.22	0.33	1.78	2.22	0.44
	2	1.94	2.09	0.15	0.76	2.09	1.33
	3	0.94	1.82	0.88	0.59	1.82	1.23
	4	0.36	1.70	1.34	0.16	1.70	1.54

It can be concluded from Table 4 that when the forecast week is 1–3, the absolute error (normalized) between the forecast value and the actual value is not >16.99 (it should be noted that the normalized absolute error is distributed between 0 and 100; the same applies below). When the region scope of the data is global or India-based, the absolute error between the forecast value and the actual value is not >3.10. Therefore, it can be considered that the transfer function model has high accuracy in forecasting the development trend of the COVID-19 pandemic.

### Analysis of forecast accuracy of the transfer function model with changes in the last point of the response sequence

#### Selection of the last point

**Figure 6:**
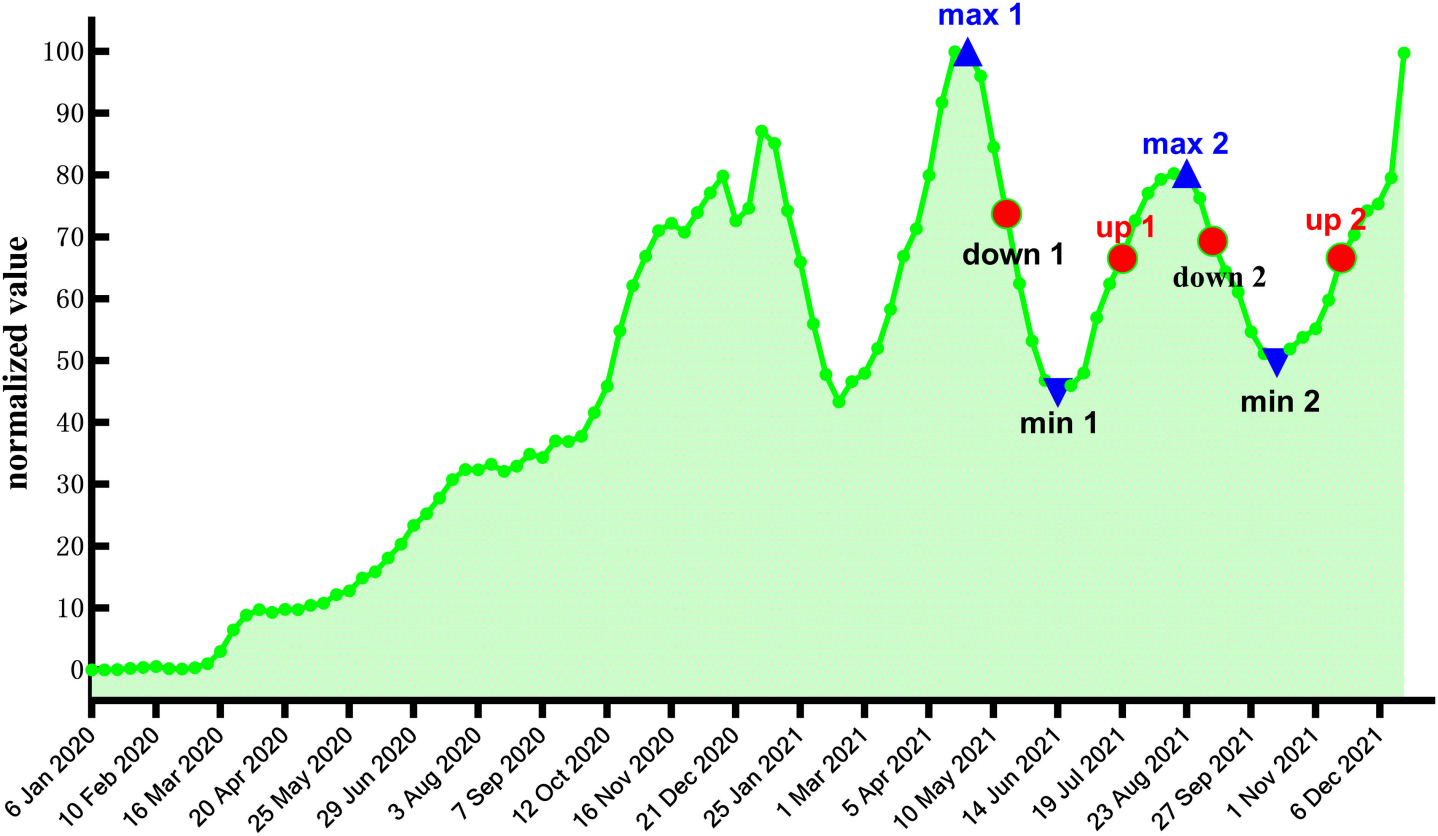
Timing diagram of weekly new confirmed cases worldwide.

Considering that the forecast accuracy of the model will be affected by changes in the position of the last point of the response sequence, we selected two midpoints of the upward trend, two midpoints of the downward trend, two maximum points, and two minimum points in the timing diagram of weekly new confirmed cases of COVID-19 worldwide as the last point (Figure 6). Then, using Google Trends of anosmia worldwide as the input sequence, we established different transfer function models for the data before different last points and forecasted the weekly number of newly confirmed cases in the following 4 weeks after the last points. Considering that the difference between the last points will lead to a change in the amount of raw data for modeling, to reduce the influence of this factor on the forecast accuracy, the dates of the eight last points are relatively close to each other.

#### Analysis of forecast accuracy when the last point is at the midpoint of the upward trend or downtrend

In this study, the number of new confirmed cases during the week of 25 July 2021 and the week of 21 November 2021 were selected as the last points, which were at the midpoint of the upward trend. Then, we used data before the cutoff date as the raw data to build the transfer function model for forecasting. The forecast results and absolute error are shown in Table 5.

**Table 5:** Forecast result when the last point is at the midpoint of the upward trend.

**Position of the last point**	**Cutoff date**	**25 July 2021**			**21 November 2021**		
	**Week**	**Forecast**	**Actual value**	**Absolute error**	**Forecast**	**Actual value**	**Absolute error**
Mid-point of the uptrend	1	69.69	72.68	3.00	71.74	70.38	1.35
	2	72.46	77.11	4.65	75.76	74.26	1.50
	3	74.43	79.35	4.92	78.80	75.34	3.46
	4	75.34	80.27	4.93	80.71	79.60	1.11

The number of newly confirmed COVID-19 cases during the week of 23 May 2021 and the week of 12 September 2021 were selected as the last points, which were at the midpoint of the downtrend. Then, we used the data before the cutoff date as the raw data to build the transfer function model for forecasting. The forecast results and absolute error are shown in Table 6.

**Table 6:** Forecast result when the last point is at the midpoint of the downward trend.

**Position of the last point**	**Cutoff date**	**23 May 2021**			**12 September 2021**		
	**Week**	**Forecast**	**Actual value**	**Absolute error**	**Forecast**	**Actual value**	**Absolute error**
Mid-point of the downtrend	1	62.64	62.49	0.15	62.06	64.40	2.34
	2	52.74	53.17	0.43	55.58	61.09	5.51
	3	44.74	46.83	2.09	50.28	54.65	4.37
	4	39.02	44.78	5.76	46.37	51.14	4.77

**Figure 7:**
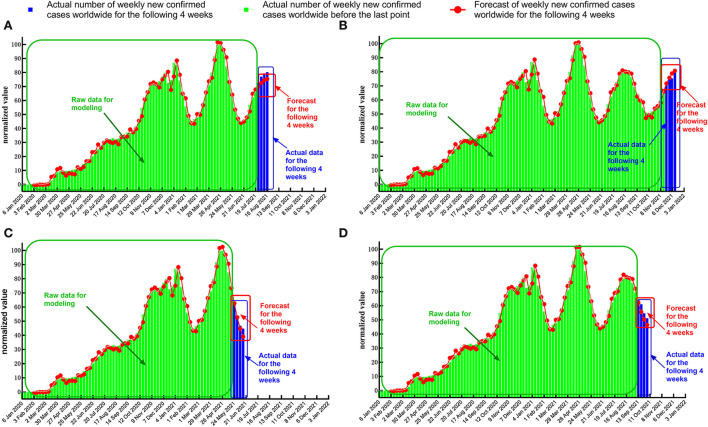
Timing diagram of forecasts and actual values when the last point of the response sequence is at the midpoint of the upward trend or downtrend. **(A)** Date of the last point: 25 July 2021 (upward trend 1). **(B)** Date of the last point: 21 November 2021 (upward trend 2). **(C)** Date of the last point: 23 May 2021 (downtrend 1). **(D)** Date of the last point: 12 September 2021 (downtrend 2).

The timing diagram of actual data and forecast value was plotted using GraphPad Prism 8.0 for data visualization (Figure 7).

#### Analysis of the forecast accuracy when the last point is at the maximum point or minimum point

In this study, the number of newly confirmed COVID-19 cases during the week of 2 May 2021 and the week of 29 August 2021 were selected as the last points, which were at the maximum points in the response sequence. Then, we used the data before the cutoff date as the raw data to build the transfer function model for forecasting. The forecast results and absolute error are shown in Table 7.

**Table 7:** Forecast result when the last point is at the maximum point.

**Position of the last point**	**Cutoff date**	**2 May 2021**			**29 August 2021**		
	**Week**	**Forecast**	**Actual value**	**Absolute error**	**Forecast**	**Actual value**	**Absolute error**
Maximum point	1	99.27	96.04	3.23	79.29	76.31	2.98
	2	96.46	84.56	11.90	77.08	69.35	7.73
	3	93.32	73.78	19.54	74.05	64.40	9.65
	4	88.49	62.49	26.00	70.65	61.09	9.56

The number of new confirmed cases in the week of 20 June 2021 and the week of 17 October 2021 were selected as the last points, which were the minimum points in the response sequence. Then, we used the data before the cutoff date as the raw data to build the transfer function model for forecasting. The forecast results and absolute error are shown in Table 8.

**Table 8:** Forecast result when the last point is at the minimum point.

**Position of the last point**	**Cutoff date**	**20 June 2021**			**17 October 2021**		
	**Week**	**Forecast**	**Actual value**	**Absolute error**	**Forecast**	**Actual value**	**Absolute error**
Minimum point	1	43.11	45.99	2.88	47.88	51.90	4.02
	2	42.90	48.03	5.13	47.25	53.80	6.55
	3	42.42	56.96	14.54	48.05	55.15	7.10
	4	42.94	62.43	19.49	50.28	59.79	9.51

**Figure 8:**
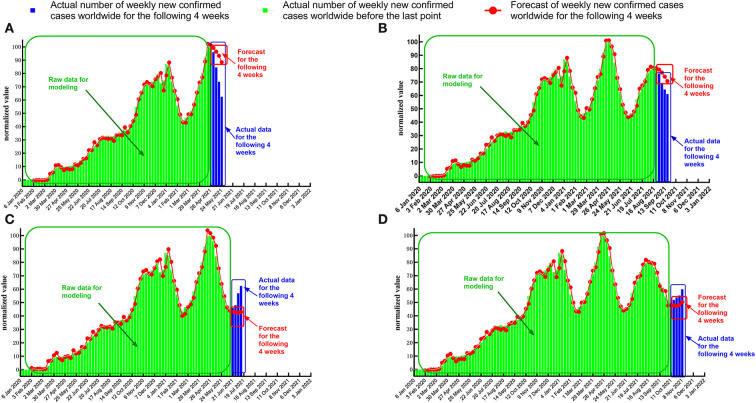
Timing diagram of forecasts and actual values when the last point of the response sequence is at the maximum or minimum point. **(A)** Date of the last point: 2 May 2021 (maximum 1). **(B)** Date of the last point: 29 August 2021 (maximum 2). **(C)** Date of the last point: 20 June 2021 (minimum 1). **(D)** Date of the last point: 17 October 2021 (minimum 2).

The timing diagram of actual data and forecast value was plotted using GraphPad Prism 8.0 for data visualization (Figure 8).

When the last point for the response sequence is at the extreme point, the standard error between the forecast value and the actual normalized data (Tables 7, 8) is more significant than in the case where the last point is at the midpoint of the uptrend or downtrend (Tables 5, 6). In some test cases (Figure 8A), the absolute error increases relatively rapidly as the number of forecast periods increases.

We found that in most test cases (Figures 8A,B,D), the transfer function model could accurately forecast the date of the inflection point of the pandemic and the trend of the response sequence in the future. Specifically, when we set the last point of the response sequence as 2 May 2021 and 29 August 2021, the transfer function model successfully forecasted that there would be a maximum point for the number of newly confirmed COVID-19 cases, which means that the intensity of the pandemic will ease after a few weeks. When we set the last point of the response sequence as 17 October 2021, the transfer function model successfully forecasted that there would be a minimum point for the number of newly confirmed cases, which means that the intensity of the pandemic will rise after a few weeks.

In summary, when the last point of the response sequence is at the extreme point, although the forecast value of the transfer function model deviates slightly from the actual value, the turning point of the pandemic can be forecasted relatively accurately. Therefore, this forecast method is of great guiding importance for accurate judgment about future trends in the COVID-19 pandemic and the deployment and adjustment of governmental prevention and control policies.

### Sensitivity analysis of the search term

To further test the prediction accuracy of the trans function model when the search term of Google Trends changed, we used “smell loss” instead of “loss of smell” as a keyword to obtain Google Trends data from 6 January 2020 to 28 November 2021 worldwide, the United States, and India. Then, we used these data as the input sequences of the model to forecast the new confirmed cases for the next 4 weeks. The prediction results and absolute errors are shown in Table 9, which showed that absolute errors were very similar to that in Table 4, which indicates that the precise forecast results we can also get when the search term was changed to “smell loss.” In conclusion, the prediction method proposed in this paper has good stability for different search terms.

**Table 9:** Prediction results and absolute errors when the search term changed to “smell loss.”

**Area**	**Search term**		**Smell loss**	
	**Week(s)**	**Forecast**	**Actual value**	**Absolute error**
Globe	1	73.11	74.26	1.15
	2	75.88	75.34	0.54
	3	77.71	79.60	1.89
	4	78.55	99.74	21.19
USA	1	34.77	44.92	10.15
	2	37.48	49.81	12.33
	3	39.19	54.04	14.85
	4	39.73	84.57	44.84
India	1	2.45	2.22	0.23
	2	2.42	2.09	0.33
	3	2.74	1.82	0.92
	4	2.93	1.70	1.23

## Discussion

### Summary of the study and comparison with contemporaneous studies

Through multivariate time-series analysis, the transfer function models forecast the development trend of COVID-19, which is of great importance in pandemic prevention and control. A few researchers have used Google Trends data to forecast trends in the development of diseases, including COVID-19 (13–15). Mavragani and Gkillas (16) applied regression analysis to Google search data on COVID-19 in the United States and found a statistically significant correlation between Google Trends and COVID-19 data. In those publications, various methods were used for analysis, including long short-term memory, random forest regression, AdaBoost algorithm, neural network autoregression, and vector error correction modeling. The conclusions indicated that the use of Google Trends data could be beneficial for forecasting and surveillance of COVID-19 spread in most countries.

However, few researchers established a forecast model using Google Trends data on smell or taste loss worldwide. Walker et al. (7) found a positive correlation between Google Trends data with loss of smell and taste using Spearman's grade correlation analysis. Henry et al. (17) used Google searches for loss of smell, taste, and fever to forecast the number of new cases of COVID-19 in Poland using linear regression. Ahmed et al. (18) used data from Pakistan to establish a linear regression model and concluded that patients' loss of smell and taste occurred roughly 2–3 weeks earlier than the time the case was diagnosed. Although relevant research has been carried out, due to the complex relationship between the number of confirmed cases and Google Trends search volume, there are still many shortcomings in linear regression. Our study improved on these by using a transfer function model in a multivariate time-series analysis, a combination of multiple regression, and time-series analysis. As a result, the accuracy of the forecast is effectively improved.

### Explanation of the regional scope of the source data selected in this study

In this study, we selected data from the United States and India, based on extensive data analysis. First, the United States and India have had many confirmed cases since the COVID-19 outbreak. Second, the Google search engine is the most widely used in the United States, India, and worldwide. Therefore, the Google Trends data from the United States and India used in this study have strong representativeness and reliability. With the popularization and improvement of Internet technology, big Internet data can be used to monitor infectious diseases earlier in many countries to prevent problems before they occur (19–21).

In the course of pandemic prevention and control in China, Internet big data has played an important role in monitoring and early warning, virus source tracking, etc. (2, 22). We have also tried to add analysis on the search volume of Chinese smell and taste keywords, but we finally concluded that there were no valid data on the loss of smell and taste in China. First, Baidu is the search engine commonly used by most netizens in China, but in the Baidu search engine, related keywords such as “loss of smell” and “loss of taste” are not included in the Baidu Index entries. According to the Baidu Index data acquisition rules, “Keywords that do not meet the inclusion criteria can be added to the Baidu Index by purchasing the right to add words. For new words created on the day, the system starts calculating and providing data services the next day and does not backtrack historical data.” According to this rule, we could not obtain online search data for keywords such as “loss of smell” and “loss of taste” during the most severe period of the pandemic in China. Second, in most cases, users use Google to search in English, Google has withdrawn from the Chinese market, and there is no relevant valid data about the pandemic in China. Based on the consideration of big data analysis, the study selected the United States and India as regions to examine.

### Explanation of error calculation in the accuracy test

In this study, we evaluated the forecast accuracy of the model by calculating the absolute error between the forecast value and the actual normalized value (abbreviated as the normalized absolute error).


$$\begin{array}{l} normalized\;absolute\;error=\;|normalized\;forecast\;value\\ \;-\;normalized\;actual\;value| \end{array}$$

First, the absolute error reflects the difference between the number of newly confirmed cases during the COVID-19 pandemic per week and the number of confirmed cases forecasted by the model. The advantage is that the error is not affected by the confirmed cases, which enables the forecast accuracy of the same model in other weeks to have a unified measurement standard.

Second, the normalized absolute error can limit the range of absolute error to 0 and 100. The advantage of this is that the error can be more intuitive for researchers in analysis. At the same time, comparing the forecast accuracies of models in different regions eliminates the effect of differences in the populations of different regions in error comparisons, which enables the forecast accuracy of the different models in different regions to have a unified measurement standard.

In summary, we used the normalized absolute error to judge model forecast accuracy.

### Discussion about the media converge

As we all know, the popularity of media coverage had a certain correlation with the search volume of Google Trends, which might affect the number of online searches in a certain period of time (21, 23), but from the overall time point of view, it could not change the overall development trend of online search volume, nor would it affect the development trend of pandemic situation. Taking India as an example, after the media reported the symptoms of COVID-19 including loss of smell and taste (around mid-March 2020), its network retrieval volume was still at a low level (data from Media Cloud). On the contrary, the media coverage had different effects on Google Trends search volume in different countries and different time periods, and it was difficult to quantify it by setting an indicator. Due to the above considerations, we did not include the impact of media coverage on the data in the calculation of the model.

### Overview of advantages and disadvantages of this study

The present study has many advantages and innovations in the selection of source data and the consideration of research methods. First, in terms of source data selection, the data from Google Trends have been widely used as raw data for infectious disease research, so its accuracy has been confirmed (24–26). Second, the time duration of the data collected in this study was sufficiently long to cover many critical and peak periods of the pandemic. The cumulative number of confirmed cases reached 180 million, and the spread regions covered nearly the whole world. Third, although media reports and public opinion have a slight impact on the retrieval volume of entries (21), these cannot significantly influence the overall increase or decrease trend of data over a long period. Therefore, the accuracy of the data still could be guaranteed. In terms of research methods, we established a transfer function model for data from different regions at different periods and tested the stability and accuracy of the model from various perspectives. Regardless of the current pandemic situation, the error in the forecast was within a smaller interval without influences on the trend of COVID-19. In addition, the turning points of the pandemic could be forecasted relatively accurately, which is of importance for the deployment and adjustment of governmental prevention and control policies.

There are also some shortcomings of this study. First, we only considered time-series analysis in this study, and the only independent variable was anosmia or ageusia according to Google Trends. Compared with some mature forecast models, this model may be somewhat simple, but the results proved that this model has sufficient forecast accuracy. Our study provides a heuristic idea to which researchers can add the variables of loss of smell or taste based on an existing mature forecast system to further improve forecast accuracy. Second, the study findings only provide a forecast but do not specify how to promptly deploy and adjust pandemic prevention and control policies.

## Conclusion

Google Trends data for smell and taste loss can help with the advanced forecasting of trends in COVID-19 infection. Worldwide, in the United States, and India, the weekly numbers of newly confirmed cases of COVID-19 lag Google Trends by 1–3 weeks, suggesting that Google Trends regarding loss of smell or taste could forecast the trend in COVID-19 infection up to 3 weeks in advance.

## Data availability statement

The original contributions presented in the study are included in the article/supplementary material, further inquiries can be directed to the corresponding author/s.

## Author contributions

JC and XR conceived and designed the initial experiments. JC, HM, JF, HZhe, HZha, RY, HG, YaZ, and KZ finalized the design of the study and performed the experiments. HM, JF, HZhe, HL, YiZ, and NS analyzed the data. JC and HM coordinated the writing of this manuscript with the contribution of JF, HZhe, HZha, and XR. All authors contributed to the article and approved the submitted version.

## Funding

This study was supported by the National Natural Scientific Foundation of China (82000960), the Fundamental Research Funds for the Central Universities (xzy012020046), and the Shaanxi Provincial Natural Science Foundation Research Program for Youth (S2021-JQ-418).

## Conflict of interest

The authors declare that the research was conducted in the absence of any commercial or financial relationships that could be construed as a potential conflict of interest.

## Publisher's note

All claims expressed in this article are solely those of the authors and do not necessarily represent those of their affiliated organizations, or those of the publisher, the editors and the reviewers. Any product that may be evaluated in this article, or claim that may be made by its manufacturer, is not guaranteed or endorsed by the publisher.
